# Dietary Patterns and Serum Gamma-Glutamyl Transferase in Japanese Men and Women

**DOI:** 10.2188/jea.JE20140158

**Published:** 2015-05-05

**Authors:** Hinako Nanri, Megumi Hara, Yuichiro Nishida, Chisato Shimanoe, Kazuyo Nakamura, Yasuki Higaki, Takeshi Imaizumi, Naoto Taguchi, Tatsuhiko Sakamoto, Mikako Horita, Koichi Shinchi, Akatsuki Kokaze, Keitaro Tanaka

**Affiliations:** 1 Department of Public Health, Showa University School of Medicine, Tokyo, Japan; 1 昭和大学医学部衛生学公衆衛生学講座公衆衛生学部門; 2 Department of Preventive Medicine, Faculty of Medicine, Saga University, Saga, Japan; 2 佐賀大学医学部社会医学講座予防医学分野; 3 St. Mary’s College Faculty of Nursing, Kurume, Fukuoka, Japan; 3 聖マリア学院大学看護学部; 4 Laboratory of Exercise Physiology, Faculty of Sports and Health Science, Fukuoka University, Fukuoka, Japan; 4 福岡大学スポーツ科学部; 5 Chikushi Office for Health, Human Services and Environmental Issues, Fukuoka Prefectural Government, Fukuoka, Japan; 5 福岡県筑紫保健福祉環境事務所; 6 Division of International Health and Nursing, Faculty of Medicine, Saga University, Saga, Japan; 6 佐賀大学医学部地域国際保健看護学講座国際保健看護学分野

**Keywords:** dietary pattern, gamma-glutamyl transferase, factor analysis

## Abstract

**Background:**

Although specific foods and nutrients have been examined as potential determinants of serum gamma-glutamyl transferase (GGT) concentrations, the relationship between dietary patterns and GGT remains unknown. The present cross-sectional study aimed to determine relationships between dietary patterns and GGT concentrations, and the effects of lifestyle factors on GGT.

**Methods:**

Relationships between dietary patterns and GGT were analyzed in 9803 Japanese individuals (3723 men and 6080 women age 40–69 years) without a history of liver diseases or elevated serum aminotransferase. We examined major dietary patterns by factor analysis of 46 items determined from a validated, short food frequency questionnaire.

**Results:**

We defined dietary patterns as healthy, Western, seafood, bread, and dessert. The healthy pattern was inversely related to GGT in men (odds ratio [OR] for highest vs lowest quartile, 0.72; 95% confidence interval [CI], 0.57–0.92; *P* < 0.01 for trend) and women (OR 0.82; 95% CI, 0.66–1.0; *P* = 0.05 for trend), whereas the seafood pattern was positively related to GGT in men (OR 1.27; 95% CI, 1.01–1.61; *P* = 0.03 for trend) and women (OR 1.21; 95% CI, 0.98–1.49; *P* = 0.05 for trend). Male-specific inverse associations with GGT were found for bread and dessert patterns (OR 0.63; 95% CI, 0.50–0.80 and OR 0.53; 95% CI, 0.41–0.68, respectively; *P* < 0.01 for both trends). Seafood or bread patterns and alcohol consumption significantly interacted with GGT in men (*P* = 0.03 and <0.01 for interaction, respectively) and between the dessert pattern and body mass index or smoking habit in women (*P* = 0.03 and <0.01, respectively, for interaction).

**Conclusions:**

Dietary patterns may be important determinants of GGT, and their possible clinical implications warrant further investigation.

## Abstract (ja)

**背景：:**

栄養素や食品は、血中γ-グルタミルトランスフェラーゼ（GGT）の潜在的な決定要因であることについてこれまでに検討されているが、食事パターンとの関連については、不明なままである。本研究の目的は、食事パターンと血中GGTとの関連および生活習慣因子による影響について検討することである。

**方法：:**

この横断研究は、肝疾患の既往歴者および血中トランスフェラーゼ高値の者等を除外した9,803名の日本人（40～69歳の男性3,723名、女性6,080名）を解析対象者とした。妥当性が検討されている46食品の食物摂取頻度調査票を用いて、因子分析により代表的な食事パターンを抽出した。

**結果：:**

代表的な食事パターンはhealthy, Western, seafood, bread, dessertが抽出された。食事パターンの因子得点4分位の最も低い群(Q1)に対する最も高い群(Q4)のGGT高値のオッズ比は、男女ともにhealthy patternにおいて有意な低下が認められ、男性で0.72（95% CI, 0.57-0.92; *P* for trend < 0.01）、女性で0.82（0.66-1.00; *P *= 0.05）であった。一方、seafood patternでは有意な上昇が認められ、男性で1.27（1.01-1.61; *P* = 0.03）、女性で1.21（0.98-1.49; *P* = 0.05）であった。また、男性のみbread pattern（OR = 0.63; 0.50-0.80; *P* < 0.01）とdessert pattern（OR = 0.53; 0.41-0.68; *P* < 0.01）で有意な低下が認められた。食事パターンと生活習慣因子の交互作用を検討した結果、男性では、seafood pattern（*P* = 0.03）およびbread pattern（*P* < 0.01）とGGT高値の関連において飲酒習慣との有意な交互作用が認められ、女性ではdessert patternとGGT高値の関連において、BMI（*P* = 0.03）または喫煙習慣（*P* < 0.01）との有意な交互作用が認められた。

**結論：:**

食事パターンは、GGTの重要な決定因子である可能性が示唆され、それらの臨床的意義についてはさらなる調査が必要である。

## INTRODUCTION

Serum γ-glutamyl transferase (GGT) is an established clinical marker of liver disease and excessive alcohol consumption.^1^ Recent studies have also suggested other significant aspects of this marker. Epidemiological studies have shown that serum GGT is related to markers of oxidative stress,^2^ and experimental findings have shown that GGT plays a crucial role in cellular antioxidant defense systems.^3^ Serum GGT concentration is also positively associated with markers of inflammation, such as C-reactive protein (CRP),^4^^,^^5^ and risk of cardiovascular disease (CVD).^6^^,^^7^

Recent epidemiological studies suggest that elevated serum GGT is associated with heavy alcohol consumption,^1^ smoking,^8^ and lower levels of physical activity.^9^ Several studies have investigated relationships between dietary factors and serum GGT concentration.^10^^–^^15^ Higher intake and blood concentrations of antioxidant vitamins and carotenoids are reportedly associated with reduced serum GGT.^11^^,^^12^^,^^15^ In addition, the proportion of energy derived from carbohydrates, sugar, and coffee intake is inversely associated with serum GGT.^10^^,^^13^^,^^14^ In contrast, high intake of dietary iron, the major source of which is heme iron in fish and meat, is associated with elevated serum GGT.^12^

Dietary patterns have recently attracted much attention in nutritional epidemiology due to the complexity of dietary exposure, the potential roles of multiple dietary components, and their interactions in health and disease.^16^ However, only one study has found an inverse association between Mediterranean diet scores and serum GGT,^17^ and to our knowledge, the relationship between dietary patterns and serum GGT concentrations in Japan has not been clarified. This cross-sectional study aimed to uncover relationships between dietary patterns and serum GGT in a Japanese population and identify interactions between dietary choices and lifestyle factors (such as alcohol consumption) on serum GGT concentrations.

## MATERIALS AND METHODS

### Study population

We analyzed baseline data from the population-based Japan Multi-Institutional Collaborative Cohort study conducted in Saga City, which aims to accumulate fundamental data about the prevention and genetics of lifestyle-related diseases, particularly cancer.^18^ The participants and methods of the Saga cohort are described in detail elsewhere.^19^^,^^20^ We mailed invitations to 61 447 residents between the ages of 40 and 69 years to participate in a baseline survey between 2005 and 2007. Of those invited, 12 078 (19.7%) agreed to participate. Ten of them later withdrew, leaving 12 068 (5078 men and 6990 women) who expressed interest in participating. Of these, 2265 were excluded due to missing information about serum GGT, aspartate aminotransferase (AST), and/or alanine aminotransferase (ALT) (*n* = 6); history of fatty liver, hepatitis, or cirrhosis (*n* = 1707); positivity for hepatitis B surface antigen (HBsAg) or hepatitis C antibody (anti-HCV) (*n* = 204); serum GGT >500 U/L or AST and/or ALT ≥50 U/L (*n* = 339); or dietary energy intake <500 or ≥3500 kcal/day (*n* = 9). Consequently, 9803 participants (3723 men and 6080 women) provided written informed consent and were enrolled in the study. The Ethics Committees at Saga University Faculty of Medicine and at Nagoya University Graduate School of Medicine approved the study protocol.

### Dietary assessment

A validated short food frequency questionnaire (FFQ)^21^^–^^24^ assessed average intake of 46 food and beverage (eg, green tea and coffee) items over the previous year, as described in an earlier report.^20^ Daily intake of staple foods (eg, rice, bread, and noodles) was estimated from six categories of frequency (essentially none, 1–3 times/month, 1–2 times/week, 3–4 times/week, 5–6 times/week, and daily) and amounts per meal (bowls for rice/noodles and slices/rolls for bread) consumed during breakfast, lunch, and supper. For the other 43 dietary items, eight categories with assigned daily consumption frequencies of essentially none (0), 1–3 times/month (0.1), 1–2 times/week (0.2), 3–4 times/week (0.5), 5–6 times/week (0.8), once/day (1), twice/day (2), and ≥3 times/day (3) were recorded.

### Blood sampling and laboratory assays

We collected venous blood at the baseline survey and sent serum samples to an external testing laboratory (SRL, Fukuoka, Japan) for analysis. Serum GGT, AST, and ALT concentrations were measured using autoanalyzers (GGT: EDC EZ scan, Tokyo, Japan; AST and ALT: Olympus AU 5431, Tokyo, Japan). Serum HBsAg and anti-HCV were assayed using a chemiluminescent immunoassay (CLIA; Dinabot, Tokyo, Japan) and a third-generation enzyme immunoassay (CLEIA; Fuji Rebio, Tokyo, Japan), respectively.

### Other baseline variables

A self-administered questionnaire ascertained demographic characteristics, lifestyle habits (including alcohol consumption and smoking status), medical history, and menopausal status (for women). A research nurse at the survey location followed up with participants whose surveys had missing or inconsistent answers. Total ethanol consumption (g/day) was estimated from reported consumption frequencies and amounts of five types of alcoholic beverages. Height (cm) and weight (kg) were measured, and body mass index (BMI) was calculated as weight (kg) divided by height (m) squared. Almost all participants (98.6%) wore a Kenz Lifecorder EX single-axis accelerometer (Suzuken Co., Ltd., Nagoya, Japan) for 10 days after the baseline survey. Physical activity levels (PALs) were calculated as total energy expenditure (kcal/day) divided by basal metabolic rate (kcal/day); the former was estimated from the accelerometer as average daily energy expenditure (excluding the initial 3 days), and the latter was calculated as basal metabolic standard^25^ × body surface area^26^ × 24 hours.

### Statistical analysis

All data were statistically analyzed separately for men and women using the SAS statistical software package Ver. 9.3 for Windows (SAS Institute, Cary, NC, USA). The intake of selected major nutrients was estimated using the SAS software based on information from the FFQ and the Standard Tables of Food Composition in Japan (fifth revised edition).^27^ Dietary patterns were derived from sex-specific factor analysis of 46 food items without alcohol consumption (daily amounts of three staple foods and daily frequencies of 43 foods and beverages) using the FACTOR Procedure of SAS. The identified factors were rotated using varimax rotation to improve their interpretability. Factor numbers were selected mainly according to eigenvalues >1, scree plots, and factor interpretability. The derived factors (dietary patterns) were labeled according to food items with high factor loadings on each factor. Factor scores for each dietary pattern (dietary pattern score) in an individual were estimated as a linear combination of standardized values for food items and standardized scoring coefficients. Each dietary pattern score was categorized into quartiles based on sex-specific distribution.

In univariate analyses, differences in means were determined using *t* tests, and differences in proportions were analyzed using chi-square tests. Associations between dietary pattern scores (categorized into quartiles, with assigned scores of 0, 1, 2, and 3) with selected characteristics at baseline were evaluated by linear regression analysis (for continuous variables) or the Mantel-Haenszel test (for categorical variables). Correlations between dietary factor scores and selected nutrients were analyzed by using Pearson’s correlation coefficients. We defined elevated serum GGT as >51 U/L for men and >33 U/L for women, in accordance with Gunter et al.^28^ Adjusted odds ratios (ORs) and 95% confidence intervals (CIs) for elevated serum GGT according to quartiles of dietary pattern scores were estimated using unconditional logistic regression analysis. Adjustments were applied for age (years), BMI (kg/m^2^), alcohol consumption (never, former, or currently consuming 0.1–22.9, 23.0–45.9, or ≥46.0 g ethanol/day), smoking (never, former, or currently smoking 1–19, 20–39, or ≥40 cigarettes/day), PAL (continuous), and menopausal status (pre- or post-menopausal) for women. Linear trends of ORs were evaluated using an ordinal variable for each dietary pattern score. We tested whether associations between dietary patterns and elevated serum GGT differed according to the selected factors of BMI, alcohol, smoking, and PAL by including an additional interaction term for the dietary pattern score and the factor of interest as ordinal variables in the model. *P* < 0.05 was considered statistically significant.

## RESULTS

Table 1 shows major food and beverage items with high factor loadings on five dietary patterns identified by sex-specific factor analysis. Details of factor loadings are defined elsewhere,^20^ although they slightly differ from actual factor loadings in this study due to different exclusion criteria of the participants. These patterns were identically labeled for men and women as healthy (high intake of vegetables, fruits other than citrus, fish and natto [fermented soybeans], or soybeans), Western (high intake of deep-fried or stir-fried foods, mayonnaise, meat, and eggs), seafood (high intake of shellfish, squid, octopus, shrimp, crab, fish roe, and fish), bread (high intake of bread, margarine, and coffee; low intake of rice and miso soup), and dessert (high intake of Western/Japanese confections and fruits).

**Table 1.** Factor-loadings of major food items^a^ on dietary patterns identified by sex-specific factor analysis in 3723 men and 6080 women

Dietary pattern	Food item	Factor loading^b^	

		Men	Women
Healthy	Carrots	0.68	0.67
	Daikon (Japanese radish)	0.66	0.58
	Green leafy vegetables	0.64	0.70
	Other green/yellow vegetables	0.63	0.72
	Mushrooms	0.62	0.62
	Other vegetables	0.61	0.69
	Potatoes	0.59	0.54
	Pumpkin	0.58	0.48
	Cabbage	0.55	0.61
	Seaweed	0.55	0.54
	Broccoli	0.52	0.40
	Fruit other than citrus	0.42	0.45
	Fish	0.39	0.29
	Natto and soybean	0.36	0.28
	Variance explained (%)	5.78	5.31
Western	Deep-fried foods	0.66	0.57
	Stir-fried foods	0.65	0.46
	Mayonnaise	0.58	0.52
	Ham/sausage/salami/bacon	0.55	0.61
	Beef or pork	0.54	0.58
	Egg	0.43	0.47
	Chicken	0.48	0.56
	Coffee	0.30	0.18
	Variance explained (%)	3.12	2.94
Seafood	Shellfish	0.68	0.43
	Squid/octopus/shrimp/crab	0.64	0.23
	Liver	0.41	0.45
	Fish roe	0.49	0.26
	Bone-edible small fish	0.40	0.52
	Fish	0.36	0.43
	Fish-paste products	0.34	0.18
	Canned tuna	0.23	0.40
	Variance explained (%)	2.32	2.73
Bread	Bread	0.71	0.73
	Rice	−0.67	−0.70
	Margarine	0.53	0.57
	Miso soup	−0.50	−0.40
	Milk	0.31	0.29
	Yogurt	0.27	0.28
	Coffee	0.30	0.39
	Green tea	−0.22	−0.30
	Variance explained (%)	2.16	2.20
Dessert	Japanese-style confections	0.64	0.60
	Western-style confections	0.59	0.55
	Citrus fruit	0.50	0.34
	Other fruit	0.50	0.32
	Peanut	0.32	0.34
	Variance explained (%)	1.97	2.06

^a^Only selected food items with high factor loadings are listed in descending order of absolute values of factor loadings in men. Details of factor loadings have been described elsewhere (reference ^20^) although they slightly differ from actual factor loadings in this study, due to different exclusion criteria.

^b^Factor loadings represent correlation coefficients between each food item and each dietary pattern score.

The dietary patterns were significantly associated with baseline characteristics (Table 2). The healthy dietary pattern was positively associated with age and alcohol consumption and inversely associated with smoking in men; in women, the healthy dietary pattern was positively associated with menopause and inversely associated with BMI, alcohol consumption, and smoking. Conversely, the Western pattern was inversely associated with age and positively with BMI, alcohol consumption, and smoking in both sexes; in women, the Western pattern was also inversely associated with menopause and positively associated with PAL. The seafood pattern was positively associated with age in both sexes, BMI and menopause in women, and alcohol consumption in men; the pattern was inversely associated with smoking in women. The bread pattern was inversely associated with age in both sexes, BMI and menopause in women, and alcohol consumption in men; it was also positively associated with alcohol consumption and smoking in both sexes and with PAL in women. The dessert pattern was positively associated with age in both sexes and with BMI and menopause in women; the pattern was inversely associated with alcohol consumption in men and smoking in both sexes.

**Table 2.** Characteristics of study subjects (3723 men and 6080 women) by quartiles of each dietary pattern score

		Age (years)		Body mass index (kg/m^2^)^a^		Current drinking^b^		Current smoking^c^		Physical activity level^d^		Postmenopausal^e^
					
		Men	Women	Men	Women	Men	Women	Men	Women	Men	Women	Women
Healthy	Q1 (low)	54.1 ± 7.8^f^	53.8 ± 8.2	23.4 ± 3.1	22.4 ± 3.3	75.4	45.8	51.5	14.9	1.453 ± 0.097	1.461 ± 0.083	59.6
	Q4 (high)	59.4 ± 7.8	57.2 ± 8.1	23.3 ± 2.8	21.9 ± 3.0	80.1	37.3	25.4	3.4	1.455 ± 0.092	1.466 ± 0.081	74.9
*P*_trend_^g^		<0.01	<0.01	0.73	<0.01	<0.01	<0.01	<0.01	<0.01	0.61	0.20	<0.01
Western	Q1 (low)	61.1 ± 6.4	59.0 ± 7.1	23.1 ± 2.9	21.9 ± 3.0	78.9	36.3	26.4	6.5	1.451 ± 0.099	1.459 ± 0.082	84.9
	Q4 (high)	52.7 ± 8.1	52.0 ± 8.1	23.4 ± 3.1	22.3 ± 3.2	80.3	45.3	45.8	10.3	1.457 ± 0.088	1.472 ± 0.081	49.7
*P*_trend_		<0.01	<0.01	0.08	<0.01	0.44	<0.01	<0.01	<0.01	0.09	<0.01	<0.01
Seafood	Q1 (low)	56.4 ± 8.3	52.2 ± 8.0	23.1 ± 2.9	22.1 ± 3.1	71.9	40.4	37.8	11.1	1.456 ± 0.095	1.467 ± 0.080	51.5
	Q4 (high)	57.2 ± 8.0	58.9 ± 7.3	23.3 ± 3.0	22.4 ± 3.1	85.4	40.1	36.9	6.5	1.451 ± 0.094	1.465 ± 0.085	69.4
*P*_trend_		0.05	<0.01	0.35	<0.01	<0.01	0.55	0.49	<0.01	0.26	0.37	<0.01
Bread	Q1 (low)	58.9 ± 7.5	57.5 ± 8.0	23.4 ± 3.0	22.4 ± 3.2	81.8	36.3	30.0	5.53	1.458 ± 0.096	1.462 ± 0.079	76.3
	Q4 (high)	56.3 ± 8.1	53.8 ± 8.2	23.2 ± 2.8	21.7 ± 2.9	76.3	45.6	36.9	10.9	1.451 ± 0.015	1.471 ± 0.086	60.4
*P*_trend_		<0.01	<0.01	0.30	<0.01	<0.01	<0.01	0.15	<0.01	0.10	<0.01	<0.01
Dessert	Q1 (low)	55.4 ± 8.4	54.1 ± 8.5	23.2 ± 2.9	22.0 ± 3.1	87.7	42.0	45.8	13.5	1.453 ± 0.094	1.469 ± 0.083	61.0
	Q4 (high)	58.5 ± 7.5	56.8 ± 7.9	23.3 ± 2.9	22.4 ± 3.0	71.6	40.4	28.3	5.5	1.456 ± 0.092	1.466 ± 0.079	73.7
*P*_trend_		<0.01	<0.01	0.87	<0.01	<0.01	0.26	<0.01	<0.01	0.51	0.53	<0.01

Q, quartile.

^a^Based on 3718 men and 6076 women.

^b^Based on 3719 men and 6074 women.

^c^Based on 3723 men and 6079 women.

^d^Calculated as total energy expenditure (kcal/d) divided by basal metabolic rate (kcal/d) in 3661 men and 6012 women.

^e^Based on 6071 women.

^f^Values are mean ± standard deviation for continuous variables and percentages for current drinking/smoking and menopausal status in the first (Q1) and fourth (Q4) quartiles of each dietary pattern score.

^g^*P* values for linear trends across quartiles (assigned ordinal numbers 0–3, respectively) of each dietary pattern score are based on linear regression analysis for continuous variables and the Mantel test for current drinking/smoking and menopausal status (only in women).

Table 3 shows Pearson’s correlation coefficients between dietary pattern scores and nutrient intake. All dietary patterns were significantly associated with the intake of almost all nutrients in both men and women. Based on major features, the healthy pattern positively correlated with dietary fiber, iron, and vitamin C, whereas the Western pattern positively correlated with monounsaturated fatty acids (MUFAs), fat, polyunsaturated fatty acids (PUFAs), n-3 PUFAs, and cholesterol. The seafood dietary pattern correlated positively with protein, cholesterol (in men), n-3 PUFAs (in men), sodium, iron, and dietary fiber (in women). The bread pattern correlated negatively with carbohydrates, energy intake (in men), and iron, and the pattern correlated positively with fat and saturated fatty acids. The dessert pattern correlated positively with vitamin C, dietary fiber, and PUFAs.

**Table 3.** Pearson correlation coefficients between each dietary pattern and daily nutrient intake in study subjects (3723 men and 6080 women)^a^

	Healthy		Western		Seafood		Bread		Dessert	
				
	Men	Women	Men	Women	Men	Women	Men	Women	Men	Women
Energy	0.13	0.24	0.22	0.30	0.09	0.08	−0.31	−0.15	0.19	0.29
Carbohydrates	0.08	0.22	0.10	0.12	−0.005	0.04	−0.40	−0.36	0.24	0.23
Protein	0.43	0.34	0.34	0.46	0.35	0.43	−0.10	0.02	0.11	0.25
Fat	0.29	0.32	0.71	0.69	0.17	0.05	0.29	0.28	0.23	0.24
Cholesterol	0.23	0.18	0.44	0.49	0.28	0.12	−0.10	0.02	0.10	0.27
SFAs	0.28	0.21	0.27	0.33	0.20	0.20	0.31	0.30	0.16	0.15
MUFAs	0.22	0.30	0.83	0.75	0.17	−0.06	0.01	0.01	0.21	0.26
PUFAs	0.33	0.39	0.69	0.60	0.20	0.11	−0.10	−0.06	0.27	0.30
n-3 PUFAs	0.36	0.38	0.54	0.51	0.25	0.14	−0.12	−0.11	0.15	0.28
Sodium	0.40	0.31	−0.001	0.07	0.22	0.34	−0.02	−0.03	0.12	0.27
Iron	0.69	0.65	0.11	0.08	0.20	0.43	−0.32	−0.26	0.22	0.24
Vitamin C	0.68	0.68	0.07	−0.05	0.07	0.26	−0.02	−0.03	0.44	0.29
Dietary fiber	0.84	0.82	0.06	0.01	0.12	0.36	0.13	0.06	0.27	0.24

MUFAs, monounsaturated fatty acids; PUFAs, polyunsaturated fatty acids; SAFs, saturated fatty acids.

^a^Correlation coefficient >0.02 or <−0.02 was considered significant.

Serum GGT levels ranged from 7 to 467 U/L (median, 34 U/L) in men and from 4 to 430 in women (median, 18 U/L), and were elevated (>51 U/L for men and >33 U/L for women) in 1012 (27.2%) men and 974 (16.0%) women. Table 4 shows multivariate-adjusted ORs and 95% CIs for elevated serum GGT according to quartiles of dietary pattern scores. The healthy pattern was inversely associated with serum GGT in men (OR for the highest vs lowest quartile, 0.72; 95% CI, 0.57–0.92; *P* < 0.01 for trend) and women (OR 0.82; 95% CI, 0.66–1.00; *P* = 0.05 for trend). The Western pattern was not significantly associated with serum GGT in either men or women (*P* = 0.16 and 0.79, respectively, for trend). The seafood pattern was positively associated with serum GGT in both men (OR 1.27; 95% CI, 1.01–1.61, *P* = 0.03 for trend) and women (OR 1.21; 95% CI, 0.98–1.49, *P* = 0.05 for trend). Associations with GGT were inverse for the bread (OR 0.63; 95% CI, 0.50–0.80; *P* < 0.01 for trend) and dessert (OR 0.53; 95% CI, 0.41–0.68; *P* < 0.01 for trend) patterns in men, but not in women (*P* = 0.89 and 0.98 for trend, respectively).

**Table 4.** Multivariate-adjusted odds of elevated serum GGT according to quartiles of each dietary pattern score

		Men (*n* = 3723)				Women (*n* = 6080)			
	
		Total *n*	Elevated GGT^a^ *n* (%)	OR^b^	95% CI	Total *n*	Elevated GGT *n* (%)	OR	95% CI
Healthy	Q1	931	273 (29.3)	1.00	(reference)	1520	277 (18.2)	1.00	(reference)
	Q2	930	268 (28.8)	0.92	(0.73–1.15)	1520	241 (15.9)	0.92	(0.75–1.12)
	Q3	932	254 (27.3)	0.84	(0.66–1.06)	1520	235 (15.5)	0.90	(0.73–1.10)
	Q4	930	217 (23.3)	0.72	(0.57–0.92)	1520	221 (14.5)	0.82	(0.66–1.00)
	*P*_trend_^c^			<0.01				0.054	
Western	Q1	931	250 (26.9)	1.00	(reference)	1520	243 (16.0)	1.00	(reference)
	Q2	930	257 (27.6)	0.97	(0.77–1.22)	1520	247 (16.3)	1.06	(0.87–1.29)
	Q3	932	246 (26.4)	0.85	(0.67–1.08)	1520	264 (17.4)	1.22	(0.998–1.49)
	Q4	930	259 (25.6)	0.86	(0.67–1.10)	1520	220 (14.5)	1.01	(0.81–1.25)
	*P*_trend_			0.16				0.79	
Seafood	Q1	930	196 (21.1)	1.00	(reference)	1520	242 (15.0)	1.00	(reference)
	Q2	932	228 (24.5)	1.10	(0.87–1.40)	1520	252 (15.6)	1.01	(0.82–1.24)
	Q3	930	283 (30.4)	1.31	(1.04–1.65)	1520	262 (16.3)	1.05	(0.86–1.30)
	Q4	931	305 (32.8)	1.27	(1.01–1.61)	1520	320 (19.9)	1.21	(0.98–1.49)
	*P*_trend_			0.03				0.051	
Bread	Q1	931	289 (31.0)	1.00	(reference)	1520	253 (16.6)	1.00	(reference)
	Q2	930	294 (31.6)	0.99	(0.80–1.24)	1520	264 (17.4)	1.11	(0.91–1.36)
	Q3	932	241 (25.9)	0.79	(0.63–0.99)	1520	232 (15.3)	0.97	(0.79–1.19)
	Q4	930	188 (20.2)	0.63	(0.50–0.80)	1520	225 (14.8)	1.03	(0.84–1.26)
	*P*_trend_			<0.01				0.89	
Dessert	Q1	931	372 (40.0)	1.00	(reference)	1520	246 (16.2)	1.00	(reference)
	Q2	930	268 (28.8)	0.78	(0.63–0.97)	1520	227 (14.9)	0.91	(0.74–1.11)
	Q3	932	215 (23.1)	0.69	(0.55–0.86)	1520	245 (16.1)	0.95	(0.78–1.16)
	Q4	930	157 (16.9)	0.53	(0.41–0.68)	1520	256 (16.8)	0.97	(0.80–1.19)
	*P*_trend_			<0.01				0.98	

CI, confidence interval; GGT, gamma-glutamyl transferase; OR, odds ratio; Q, quartile.

^a^Elevated GGT represents >51 U/L in men and >33 U/L in women.

^b^Adjusted for age (years), body mass index (kg/m^2^), alcohol consumption (never, former, or current drinker consuming <23, 23.0–45.9, or ≥46 g ethanol/d), smoking (never, former, or current smoker consuming 1–19, 20–39, or ≥40 cigarettes/d), physical activity level (continuous), and menopausal status (only in women).

^c^Based on multiple logistic regression analysis assigning ordinal numbers 0–3 to quartile categories of each dietary pattern.

We further analyzed interactions between dietary patterns and BMI, alcohol consumption, smoking, and PAL on serum GGT. Interactions were significant between alcohol consumption and the seafood or bread patterns in men (Table 5). No other combinations for interaction were statistically significant (*P* > 0.1; data not shown). Upward and downward OR trends for the seafood (*P* < 0.01 for trend) and bread (*P* < 0.01 for trend) patterns were observed in men who consume alcohol (*P* = 0.50 and 0.55 for trend, respectively), but not in those who refrain from consuming alcohol (*P* = 0.03 and <0.01 for interaction, respectively).

**Table 5.** Multivariate-adjusted odds of elevated serum GGT according to quartiles of seafood or bread pattern score in nondrinking or drinking men

		Nondrinkers^a^		Drinkers^a^		Nondrinkers		Drinkers	
			
		Total *n*	Elevated GGT^b^ *n* (%)	Total *n*	Elevated GGT *n* (%)	OR^c^	95% CI	OR^c^	95% CI
Seafood	Q1	261	28 (10.7)	668	167 (25.0)	1.00	(reference)	1.00	(reference)
	Q2	197	27 (13.7)	734	201 (27.4)	1.21	(0.66–2.19)	1.09	(0.86–1.40)
	Q3	155	17 (11.0)	773	266 (34.4)	0.96	(0.49–1.88)	1.63	(1.29–2.06)
	Q4	136	12 (8.8)	795	293 (36.9)	0.79	(0.38–1.64)	1.71	(1.36–2.16)
*P*_trend_^d^						0.50		<0.01	
*P*_interaction_^e^						0.03			

Bread	Q1	169	16 (9.5)	762	273 (35.8)	1.00	(reference)	1.00	(reference)
	Q2	164	23 (14.0)	765	271 (35.4)	1.15	(0.71–3.07)	0.98	(0.78–1.21)
	Q3	197	16 (8.1)	733	224 (30.6)	0.86	(0.40–1.86)	0.76	(0.61–0.95)
	Q4	219	29 (13.2)	710	159 (22.4)	1.40	(0.70–2.79)	0.50	(0.40–0.64)
*P*_trend_						0.55		<0.01	
*P*_interaction_						<0.01			

CI, confidence interval; GGT, gamma-glutamyl transferase; OR, odds ratio; Q, quartile.

^a^Nondrinkers include never and former alcohol drinkers, and drinkers denote current alcohol drinkers.

^b^Elevated GGT represents >51 U/L.

^c^Adjusted for age (years), body mass index (kg/m^2^), alcohol consumption (never or former drinking for nondrinkers; consumption of <23, 23.0–45.9, or ≥46 g ethanol/d for drinkers), smoking (never, former, or current smoker consuming 1–19, 20–39, or ≥40 cigarettes/d), and physical activity level (continuous).

^d^Based on multiple logistic regression analysis assigning ordinal numbers 0–3 to quartile categories of each dietary pattern.

^e^Interaction between each dietary pattern score (ordinal variable) and drinking status (2 categories) on elevated serum GGT.

Table 6 shows significant interactions between the dessert pattern and BMI or smoking in women, and a downward trend in OR (although unclear in women in general) among women with BMI ≥25 kg/m^2^ (*P* = 0.07 for trend) or women who currently smoke (*P* < 0.01 for trend), but not for women with BMI <25 kg/m^2^ (*P* = 0.19 for trend; *P* = 0.03 for interaction) or women who do not smoke (*P* = 0.26 for trend; *P* < 0.01 for interaction).

**Table 6.** Multivariate-adjusted odds of elevated serum GGT according to quartiles of dessert pattern score in women with BMI <25 kg/m^2^ or ≥25 kg/m^2^ and in nonsmoking or smoking women

Dessert	BMI <25 kg/m^2^		BMI ≥25 kg/m^2^		BMI <25 kg/m^2^		BMI ≥25 kg/m^2^	
			
	Total *n*	Elevated GGT^a^ *n* (%)	Total *n*	Elevated GGT *n* (%)	OR^b^	95% CI	OR^b^	95% CI
Q1	1292	177 (13.7)	226	69 (30.5)	1.00	(reference)	1.00	(reference)
Q2	1300	174 (13.4)	220	53 (24.1)	0.99	(0.79–1.25)	0.66	(0.43–1.03)
Q3	1273	189 (14.9)	246	56 (22.8)	1.06	(0.84–1.33)	0.68	(0.44–1.05)
Q4	1267	200 (15.8)	252	56 (22.2)	1.14	(0.91–1.43)	0.63	(0.41–0.98)
*P*_trend_^c^					0.19		0.07	
*P*_interaction_^d^					0.03			

Dessert	Nonsmokers^e^		Smokers^e^		Nonsmokers		Smokers	
			
	Total *n*	Elevated GGT *n* (%)	Total *n*	Elevated GGT *n* (%)	OR^b^	95% CI	OR^b^	95% CI

Q1	1315	196 (14.9)	205	50 (24.4)	1.00	(reference)	1.00	(reference)
Q2	1387	202 (14.6)	133	25 (18.8)	0.95	(0.76–1.18)	0.79	(0.44–1.42)
Q3	1423	231 (16.2)	96	14 (14.6)	1.04	(0.84–1.29)	0.52	(0.26–1.06)
Q4	1436	248 (17.3)	84	8 (9.5)	1.09	(0.89–1.35)	0.29	(0.12–0.72)
*P*_trend_					0.26		<0.01	
*P*_interaction_					<0.01			

BMI, body mass index; CI, confidence interval; GGT, gamma-glutamyl transferase; OR, odds ratio; Q, quartile.

^a^Elevated GGT represents >33 U/L.

^b^Adjusted for age (years), BMI (kg/m^2^), alcohol consumption (never, former, or current drinker consuming <23, 23.0–45.9, or ≥46 g ethanol/d), smoking (never, former, or current smoker consuming 1–19, 20–39, or ≥40 cigarettes/d; for nonsmokers, never or former smoking; for smokers, consumption of 1–19, 20–39, or ≥40 cigarettes/d), and physical activity level (continuous).

^c^Based on multiple logistic regression analysis assigning ordinal numbers 0–3 to quartile categories of dessert pattern score.

^d^Interaction between dessert pattern score (ordinal variable) and BMI or smoking status (2 categories) on elevated serum GGT.

^e^Nonsmokers include never and former smokers, and smokers denote current smokers.

## DISCUSSION

This large population-based cross-sectional study uncovered an inverse association between serum GGT and the healthy dietary pattern and a positive association between serum GGT and the seafood pattern in both Japanese men and women. Inverse associations between serum GGT and bread and dessert dietary patterns were notably male-specific. Our analyses of interactions between dietary patterns and selected lifestyle factors on serum GGT, followed by stratified analyses of significant interactions, identified a positive association with the seafood pattern and an inverse association with the bread pattern in men who consume alcohol but not in men who refrain from consuming alcohol. We also found an inverse association between the dessert pattern and serum GGT in women who were overweight/obese or who smoked (though the association was not significant in women overall). To our knowledge, these associations between serum GGT and dietary patterns, as well as the above interactions between dietary patterns and lifestyle factors on serum GGT, have not been previously reported.

Vegetables and fruits are major sources of vitamins, minerals, dietary fiber, and other bioactive components. High intake of vegetables and fruits rich in antioxidants is associated with decreased serum GGT.^11^^,^^12^ A study of 3146 young female adults in the United States revealed an inverse association between the highest intake of vitamin C, β-carotene, folate, and fiber with serum GGT.^12^ Only one cross-sectional study (the ATTICA study^17^) has associated a higher score for the Mediterranean diet (high intake of vegetables, fruit/nuts, fish, cereals, and legumes) with lower serum GGT. Our present findings of an inverse association between the healthy or dessert pattern and serum GGT are consistent with these previous results.

The positive association between the seafood pattern and serum GGT in this study might be unexpected because accumulating evidence suggests that high intake of fish and fish oils containing long-chain n-3 PUFAs, such as eicosapentaenoic and docosahexaenoic acids, is associated with a reduced risk of chronic disease.^29^ However, the findings from studies of associations between n-3 PUFAs and liver disease have been inconsistent.^30^^,^^31^ The seafood pattern score in the present study was significantly and positively correlated with estimated cholesterol and iron (Table 3). One cross-sectional study suggested that higher iron intake might lead to elevated serum GGT,^12^ although there have been no reports on the association of cholesterol intake and serum GGT. High intake of these nutrients could lead to higher serum GGT, which might partly explain the positive association. However, the association observed in the present study did not change after adjustment for cholesterol and iron in men and women. In relation to the interaction between the seafood pattern and alcohol intake on serum GGT, Patere et al found that alcohol-induced oxidative stress is significantly exacerbated in the presence of PUFAs and iron,^32^ which might explain the above interaction, assuming that such oxidative stress leads to elevated serum GGT.^2^

Dietary patterns similar to the bread pattern in the present study (such as Westernized breakfast), have been associated with a lower risk of metabolic syndrome, high blood pressure, and decreased concentrations of CRP and hemoglobin A1c in general populations,^20^^,^^33^^,^^34^ although these patterns have not been examined as potential determinants of serum GGT. The intriguing finding of lower serum GGT concentrations being consistently associated with coffee intake^14^ conferred a moderate factor loading on the bread pattern of 0.30 for men and 0.39 for women in the present study. Tanaka et al found a progressively stronger inverse association between coffee and serum GGT with increasing alcohol consumption,^14^ which also resembles the interaction between the bread pattern and alcohol effects observed in the present study. The protective association between the bread pattern and serum GGT might reflect the beneficial effects of some foods/beverages (such as coffee) or nutrients that correlate with the bread pattern. Such candidate dietary factors might have male-specific effects on serum GGT, since corresponding associations or interactions were not evident in women.

Previous studies have identified inverse associations between serum GGT and the intake of sugar and confectionery^13^ and found that sucrose intake is inversely but insignificantly related to serum GGT.^12^ Confections rich in sugar or sucrose conferred high factor loadings on the dessert pattern in the present study, so they might mediate the inverse association between the dessert pattern and serum GGT apart from fruit intake, although the mechanism remains to be elucidated. Notably, this inverse association was similar in men, overweight/obese women, and women who smoked. Both obesity and smoking are positive predictors of serum GGT,^35^ and some detrimental effects of both factors on serum GGT might be diminished by following the dessert pattern, particularly in women.

This study has several methodological limitations. One is the cross-sectional design, which makes it difficult to determine whether or not the observed associations are causal. The participation rate in this study was relatively low (about 20%), which may have led to selection bias. However, the geometric means of serum GGT levels (37 U/L for men and 20 U/L for women) were similar to those found in a previous study.^36^ In an attempt to minimize the possibility of reverse causality, we excluded participants who might have changed their dietary habits due to having a history of liver disease, positive hepatitis virus markers, or elevated aminotransferase concentrations. Other diseases, such as cancer, cardiovascular disease, and diabetes, can also affect serum GGT. However, the exclusion of participants with a history of cancer (*n* = 497), cardiovascular disease (*n* = 253), and diabetes mellitus (*n* = 428) did not change our findings. Elevated serum GGT itself might have influenced dietary habits if the study participants knew their current GGT. If knowing such information had led to choosing healthier foods, such as vegetables, fruits, and fish, then the strength of the inverse association between the healthy pattern and GGT would have been underestimated, whereas that of the positive association between the seafood pattern and GGT would have been overestimated. Follow-up studies are needed to address this issue. Our findings might have been affected by potential confounding factors, such as socioeconomic status (including levels of education and occupation). However, additional adjustment for socioeconomic factors did not affect our results. Finally, our factor analysis was limited in terms of subjectivity in determining and labeling dietary patterns, which may cause difficulties in extrapolating the present findings to other populations.

In conclusion, this cross-sectional study of middle-aged and older Japanese men and women showed that healthy, seafood, bread, and dessert dietary patterns are potential determinants of serum GGT, particularly in men. Whether or not these dietary patterns have clinical implications in the development or prevention of liver disease deserves further investigation.

## ONLINE ONLY MATERIAL

Abstract in Japanese.
