# Supplementary material for: Dietary Patterns and Serum Gamma-Glutamyl Transferase in Japanese Men and Women
Source: J Epidemiol. 2015 May 5;25(5):378–86. doi: 10.2188/jea.JE20140158 (PMC4411237; doi:10.2188/jea.JE20140158)
Supplement: Abstract in Japanese. [file je-25-378-s001.pdf]

食事パターンとγ-グルタミルトランスフェラーゼとの関連

南里妃名子[1]、原めぐみ[2]、西田裕一郎[2]、島ノ江千里[2]、中村和代[3]、桧垣靖樹[4]、今泉猛[2]、田口尚人[2]、坂本龍彦[5]、堀田美加子[2]、新地浩一[6]、小風暁[1]、田中恵太郎[2]

[1]昭和大学医学部衛生学公衆衛生学講座公衆衛生学部門
[2]佐賀大学医学部社会医学講座予防医学分野
[3]聖マリア学院大学看護学部
[4]福岡大学スポーツ科学部
[5]福岡県筑紫保健福祉環境事務所
[6]佐賀大学医学部地域国際保健看護学講座国際保健看護学分野

背景：栄養素や食品は、血中γ-グルタミルトランスフェラーゼ（GGT）の潜在的な決定要因であることについてこれまでに検討されているが、食事パターンとの関連については、不明なままである。本研究の目的は、食事パターンと血中GGTとの関連および生活習慣因子による影響について検討することである。
方法：この横断研究は、肝疾患の既往歴者および血中トランスフェラーゼ高値の者等を除外した9,803名の日本人（40～69歳の男性3,723名、女性6,080名）を解析対象者とした。妥当性が検討されている46食品の食物摂取頻度調査票を用いて、因子分析により代表的な食事パターンを抽出した。
結果：代表的な食事パターンはhealthy, Western, seafood, bread, dessertが抽出された。食事パターンの因子得点4分位の最も低い群(Q1)に対する最も高い群(Q4)のGGT高値のオッズ比は、男女ともにhealthy patternにおいて有意な低下が認められ、男性で0.72（95% CI, 0.57-0.92; P for trend < 0.01）、女性で0.82（0.66-1.00; P = 0.05）であった。一方、seafood patternでは有意な上昇が認められ、男性で1.27（1.01-1.61; P = 0.03）、女性で1.21（0.98-1.49; P = 0.05）であった。また、男性のみbread pattern（OR = 0.63; 0.50-0.80; P < 0.01）とdessert pattern（OR = 0.53; 0.41-0.68; P < 0.01）で有意な低下が認められた。食事パターンと生活習慣因子の交互作用を検討した結果、男性では、seafood pattern（P = 0.03）およびbread pattern（P < 0.01）とGGT高値の関連において飲酒習慣との有意な交互作用が認められ、女性ではdessert patternとGGT高値の関連において、BMI（P = 0.03）または喫煙習慣（P < 0.01）との有意な交互作用が認められた。
結論：食事パターンは、GGTの重要な決定因子である可能性が示唆され、それらの臨床的意義についてはさらなる調査が必要である。

キーワード：食事パターン、GGT、横断研究
